# Flavor Characterization of Animal Hydrolysates and Potential of Glucosamine in Flavor Modulation

**DOI:** 10.3390/foods10123008

**Published:** 2021-12-04

**Authors:** Kathrine H. Bak, Sandra S. Waehrens, Yu Fu, Ching Yue Chow, Mikael A. Petersen, Jorge Ruiz-Carrascal, Wender L. P. Bredie, René Lametsch

**Affiliations:** 1 Department of Food Science, Faculty of Science, University of Copenhagen, Rolighedsvej 26, 1958 Frederiksberg, Denmark; sandrasn@food.ku.dk (S.S.W.); chingyuechow@food.ku.dk (C.Y.C.); map@food.ku.dk (M.A.P.); jorgeruiz@food.ku.dk (J.R.-C.); wb@food.ku.dk (W.L.P.B.); rla@food.ku.dk (R.L.); 2 Institute of Food Safety, Food Technology and Veterinary Public Health, University of Veterinary Medicine Vienna, Veterinärplatz 1, 1210 Vienna, Austria; 3 College of Food Science, Southwest University, No.2 Tiansheng Road, Beibei District, Chongqing 400715, China

**Keywords:** hydrolysates, sensory, volatile compounds, glucosamine, Maillard reaction, GC-MS

## Abstract

Bovine (meat and heart) and porcine (hemoglobin and plasma) raw materials were hydrolyzed by Protease A (both endo- and exopeptidase activity), with or without glucosamine added during the enzyme inactivation step. Hydrolysates were characterized via peptide analysis (yield, UV- and fluorescence scanning spectroscopy, and peptide size distribution via size exclusion chromatography), sensory evaluation, and volatile compound analysis via gas chromatography mass-spectrometry (GC-MS) to determine if glucosamine-induced Maillard reaction improved taste and flavor. Porcine hemoglobin produced the most flavor-neutral hydrolysate, and could expectedly have the broadest application in food products. Both bovine meat and -heart hydrolysates were high in umami, and thereby good candidates for savory applications. Porcine plasma hydrolysate was high in liver flavor and would be suitable for addition to certain meat products where liver flavor is desirable. All hydrolysates had low perceived bitterness. Glucosamine-induced Maillard reaction had just a minor influence on the sensory profile via an increased perception of sweet taste (*p* = 0.038), umami taste (*p* = 0.042), and yolk flavor (*p* = 0.038) in the hydrolysates, irrespective of raw material. Glucosamine addition had a statistically significant effect on 13 of 69 volatiles detected in the hydrolysates, but the effect was minor and raw material-specific.

## 1. Introduction

By-products from the meat industry are currently underutilized, and are used mostly in the production of low-value products such as animal feed, pet food, and fertilizer [1], or may even be disposed of as waste [2]. However, these products are rich in high-quality protein [3], meaning that there is potential for the production of value-added ingredients, e.g., in the functional foods market [4]. At the same time, the global demand for high protein foods has increased in recent years [5], signifying the potential from both an economic and a sustainability point of view of improving the utilization of the by-products that are a consequence of the meat industry.

Examples of by-product valorization include blood and organs [2], however low-value meat cuts also have valorization potential. Enzymatic hydrolysis is an efficient way of extracting amino acids and peptides from a protein-containing raw material [4]. Problems with the bitterness of protein hydrolysates have previously been reported [6], though, in a previous study [7], Fu et al. found that enzymatic hydrolysates from meat and plasma had only minor issues with regard to bitterness compared to other types of hydrolysates, e.g., casein hydrolysates [6]. Furthermore, enzymatic hydrolysis leads to the development of a number of volatile compounds, including Maillard and lipid oxidation products [8]. A number of these volatile compounds may lead to off-flavors, while others may have an advantageous effect, depending on the application of the hydrolysate.

Purposely inducing a Maillard reaction is a way of potentially improving hydrolysate characteristics. Glucosamine is an amino sugar possessing salt-sour-bitter taste characteristics [9]. Nonetheless, glucosamine is an appropriate reducing sugar to utilze in order to induce a Maillard reaction in meat-related products as this increases neither the bitterness nor the sweetness of the glycated hydrolysate. The successful application of glucosamine in the glycation of poultry protein isolate provides evidence to support this suggestion [10]. The induced Maillard reaction between hydrolysates and sugar has also proved successful in regard to soy protein hydrolysates with xylose, resulting in both reduced bitterness and an increase in desirable odors and umami [11], or flavor enhancing properties in consommé soup [12]. Similarly, Maillard reaction products formed from the glycation of sunflower protein hydrolysate with xylose and cysteine had a meat-like flavor and flavor enhancing properties [13]. The positive effects are largely credited to a high content of so-called Maillard peptides, which are peptides in the region of 1 to 5 kDa [12,13,14], and to the presence of cysteine [13], which has long been known to serve as an important precursor of meat flavor compounds in reaction with carbohydrates [15]. A carefully controlled induced Maillard reaction can also serve as a way of enhancing the functional properties of a protein [15].

Our previous study deemed the enzyme Protease A, which contains a combination of endopeptidase and exopeptidase activity, to be the best choice overall out of ten enzymes that were investigated with regard to the production of high yield hydrolysates with a relatively low bitterness, from both bovine meat and porcine plasma [7]. For this reason, Protease A was the enzyme of choice in this study. Furthermore, simultaneous studies performed in our lab with an internal sensory panel have indicated that the induction of a Maillard reaction by the addition of a small amount of glucosamine to porcine hydrolysates may lead to an improvement in taste and flavor without having a detrimental effect on the color of the hydrolysate [16].

Consequently, the aim of this work was to characterize hydrolysates from bovine (minced meat and heart) and porcine (hemoglobin and plasma) sources with or without added glucosamine based on peptide size distribution and spectral characteristics, sensory evaluation by a trained panel, and volatile compound analysis via gas chromatography-mass spectrometry (GC-MS). The four raw materials were chosen for their value-adding potential, based on Danish market conditions and on their availability in food grade form. The hypothesis is that the results will provide a reliable description of the animal hydrolysates, which can be used to suggest suitable, characteristic-dependent applications. Additionally, this study should serve as confirmation of our previous results [16], which showed that the addition of glucosamine during the enzyme inactivation step of hydrolysis presents a novel way of promoting a Maillard reaction in hydrolysates, whereby the overall production time is reduced.

## 2. Materials and Methods

### 2.1. Hydrolysate Overview and Preparation

Selection of the raw materials and the protease for the present study was based on previous studies including a sensory screening of the raw materials and ten different enzymes [7,17]. Accordingly, bovine meat, bovine heart, porcine hemoglobin, and porcine plasma, all collected in a hygienic way (i.e., food grade), were subjected to enzymatic hydrolysis using the food grade enzyme Protease A for 5 h, followed by glucosamine-induced Maillard reaction as described in detail below. An overview of the produced hydrolysates is given in Supplementary Table S1.

Minced bovine meat (21.4% protein; low price, i.e., prepared from lower value cuts) and bovine heart (18.2% protein) were purchased at a local supermarket (Inco, Copenhagen, Denmark). Porcine hemoglobin (25.2% protein) and porcine plasma (6.2% protein) were kindly supplied by DAT-Schaub A/S (Copenhagen, Denmark). Food grade glucosamine (>99.8% purity) was purchased from Blackburn Distributions Ltd. (Nelson, UK). Protease A 2SD (100,000 U/g), with both endopeptidase and exopeptidase activities, was donated by Amano Enzyme Inc. (Nagoya, Japan). All analytical reagents were purchased from Sigma-Aldrich (Steinheim, Germany). The raw materials were stored at −20 °C until further processing. The protein content of the raw materials (noted above) was determined by the AOAC method using Kjeldahl analysis [18]. 

The enzymatic hydrolysates were prepared in a pilot plant reserved for food grade materials by hydrolysis with Protease A at a concentration of 0.5% *w*/*w* for 5 h at pH 7.0 and 50 °C. The procedure was conducted according to that described by Fu et al. [7,17]. Bovine minced meat and heart were hydrolyzed according to the method described for minced meat [7], with the heart first being minced by passing it through a grinder once (Scharfen Meat Mincer model X 70, Witten, Germany) with a 3 mm plate.

For half the hydrolysate preparations, food grade glucosamine was added in a ratio of 1:10 relative to the protein content of the raw material. This addition occurred at the beginning of the heating step for enzyme inactivation (85 °C for porcine plasma hydrolysates and 90 °C for the remaining three hydrolysates). After cooling, the supernatant of each of the eight hydrolysates (four raw materials, with or without glucosamine) was kept frozen at −20 °C until further analysis.

### 2.2. Sensory Evaluation

#### 2.2.1. Sensory Panel

A descriptive sensory analysis of the hydrolysates was conducted by ten trained panelists (nine women and one man, with an age range of 22–47) selected from the trained external sensory panel of the Department of Food Science at the University of Copenhagen, Denmark. Subjects were paid for their participation. When initially becoming panel members, the subjects underwent a selection process with subsequent training on the basic tastes as well as other sensory acuities. Prior to this project, the panel members had participated in the descriptive analysis of other food types.

#### 2.2.2. Sample Preparation

Hydrolysates were normalized with water to a protein content of 1% for sensory profiling. Preparation of hydrolysate samples for sensory analysis was done by pouring 25 mL portions into 30 mL black polypropylene cups with lids. Each sample was blinded with three-digit random numbers. The samples were stored for two hours at 14 °C to obtain a serving temperature of 14 °C.

#### 2.2.3. Sensory Vocabulary Development and Profiling

The descriptive sensory analysis consisted of two training sessions with sensory vocabulary development and training on scale use, followed by two sensory profiling sessions. Each session was scheduled to two hours, which was sufficient due to the experience of the panel members and the nature of this project. During the first training session, the sensory panel generated the sensory vocabulary to describe the variation between the hydrolysates based on panel consensus (see Table 1). During the second training session, reference materials were provided to increase the panelists’ cognitive clarity towards the developed attributes. The sensory panel was also trained in assessing intensity on a 15 cm-line scale anchored from ‘not at all’ to ‘very much’. The reference materials were used to support training on sensory intensity scores on the scale. The final sensory vocabulary including definition with reference materials is shown in Table 1 and included 17 sensory descriptors.

For the sensory profiling, the panel simultaneously evaluated the hydrolysates in a complete block design with three blocks over the two profiling days, resulting in triplicate evaluations of each sample. Within each block, the samples were served in a random monadic sequence to reduce any bias related to the presentation order and carry-over effect. The intensities of the attributes were rated on the 15 cm-line scale going from “not at all” to “very much”. The panel was provided with sparkling water, and cold and lukewarm water to rinse their palates. Data were collected using the Fizz Acquisition (Version2.50B, Biosystèmes, Couternon, France). The conditions for the sensory descriptive analysis were in accordance with the guidelines in ISO 13299:2003 [19].

### 2.3. Peptide Characterization

All determinations were performed in triplicate and the results reported as the average value.

#### 2.3.1. Yield

The yield of the hydrolysates was determined as described by [7] using oven heating at 105 °C until the constant weight and subsequent calculation of the yield as the percentage of dry matter content in the supernatants relative to the dry matter content prior to centrifugation.

#### 2.3.2. Spectral Characteristics of Glycated Hydrolysates

UV and fluorescence scanning were employed to monitor the formation of intermediate Maillard reaction compounds and melanoidins from browning.

The extent of the glycation of protein hydrolysates was determined by scanning the absorbance spectra of the samples (approximately 5 mg/mL) from each treatment from 200 nm to 500 nm using a Helios Omega UV-Visible spectrophotometer (Thermo Scientific, Loughborough, UK). The formation of intermediate Maillard reaction products and melanoidins during the Maillard reaction was monitored by UV scanning at 320 and 420 nm [16,20,21].

The fluorescence spectra of native and glycated hydrolysates (approximately 5 mg/mL) were determined using a SpectraMax multi-mode microplate reader (Molecular Devices, Sunnyvale, CA, USA). The excitation wavelength was fixed at 347 nm, while emission wavelengths were fixed at 400–600 nm for the detection of fluorescent products generated by the Maillard reaction [10,16].

#### 2.3.3. Peptide Size Distribution of Native and Glycated Hydrolysates

The peptide size distribution of native and glycated hydrolysates was measured according to our previously reported method [7]. The peptide size distribution of samples was analyzed by size exclusion chromatography under isocratic conditions using a Phenomenex BioSep™ SEC-S2000 column (300 mm × 4.6 mm) (Torrance, CA, USA) on an ultra-high performance liquid chromatography (UHPLC) system (Thermo Scientific Dionex Ultimate 3000, Denmark). From each sample (1 mg/mL), 10 μL were injected, eluted isocratically (the buffer being 30% acetonitrile containing 0.1% trifluoroacetic acid) at a flow rate of 0.5 mL/min, and monitored at 214 nm.

Molecular weight (MW) distribution ranges were divided into fractions, including >10 kDa, 5–10 kDa, 1–5 kDa, 0.5–1 kDa, and <0.5 kDa. As was the case in our previous study [7], this was achieved by plotting a calibration curve using Trp (204 Da), GLV (287 Da), SGNIGFPGPK (1114 Da), insulin (5700 Da) and myoglobin (17,600 Da) as standards, and calculating MW as logMW = −0.6086t + 6.9381, where MW is the molecular weight in Da and t denotes the elution time in minutes.

### 2.4. Volatile Compound Analysis by GC-MS

Hydrolysates were used undiluted for GC-MS. Dynamic headspace sampling and the subsequent GC-MS were completed in duplicate for each sample using 20 mL of hydrolysate with 1 mL of 5 mg/L 4-methyl-1-pentanol added as internal standard. The procedure was as described in our previous studies [8,17]. In brief, the collection of the volatiles onto 200 mg Tenax-TA traps with mesh size 60/80 (Markes International, Llantrisant, UK) was made at 37 °C for 60 min. An automatic thermal desorption unit (TurboMatrix 350, Perkin Elmer, Shelton, CT, USA) was used to desorb the volatiles, followed by separation on a ZB-Wax capillary column of 30 m length, 0.25 mm internal diameter, and 0.50 µm film thickness on a gas chromatograph-mass spectrometer (7890A GC-system interfaced with a 5975C VL MSD with Triple-Axis detector (Agilent Technologies, Palo Alto, CA, USA)). The column temperature program and other gas chromatograph and mass spectrometer details were as described in [8].

### 2.5. Data Analysis

Whether or not the addition of glucosamine and the type of raw material had an overall influence on the sensory profile of the hydrolysates required exploration and, as such, we conducted a linear analysis of the variance (ANOVA) including the addition of glucosamine (+/−) and raw material (bovine meat, bovine heart, porcine hemoglobin, porcine plasma) as main effects, including their interaction. The significance level was set to 5%. The next priority was to identify sensory sample differences. Thus, a sample effect was tested on each sensory attribute by mixed linear ANOVA model with a sample as fixed factor and panelist, replicate, sample-panelist interactions, and sample-replicate interactions as random factors using the lmerTest package [20] in R v3.5.2 (R Development Core Team). The significance level was set to 5%. Post hoc pairwise comparisons were made by Tukey’s HSD test (*p* < 0.05).

For the analysis of the GC-MS data, the peak areas relative to the internal standard and mass spectra were extracted from the chromatograms using the PARAFAC2 based software PARADISe [21] and mass spectra were identified using the NIST05 database. Volatile compound identifications were verified by comparison with retention indices (RI) of either authentic reference compounds or retention indices from the available literature (tentative identification).

ANOVA on the results of the GC-MS analysis was performed using JMP 13.0.0 (SAS Institute Inc., Cary, NC, USA) and least square means were compared by Tukey’s test (*p* ≤ 0.05).

## 3. Results and Discussion

### 3.1. Sensory Characterization

Unsurprisingly, the linear ANOVA showed that the raw material type significantly (*p* < 0.05) changed the sensory profile of the hydrolysates as significant effects were found for all the sensory attributes investigated. However, the addition of glucosamine had just a minor influence on the sensory profile, with significant effects found only for umami taste (*p* = 0.042), sweet taste (*p* = 0.038), and yolk flavor (*p* = 0.038).

For a better understanding of sample differences, the sample effect was analyzed via mixed linear ANOVA. A significant sample effect (*p* < 0.05) was found for all sensory attributes. After an inspection of the pairwise comparisons (Tukey’s HSD test), the significant effects could be explained by the dominant impact of raw material on the sensory profile of the hydrolysates. Pairwise comparison of samples with and without glucosamine within each raw material did not find any significant difference between these with regard to umami taste, sweet taste, and yolk flavor. This can probably be explained by the fact that the significance levels for the attributes were very close to 0.05 and nearly non-significant in the model testing overall, hence the minimal effect of the addition of glucosamine. However, a systematic increase in intensity levels for these attributes is visible for hydrolysates with added glucosamine irrespective of the raw material (Table 2).

As mentioned, raw material type had a significant influence on the sensory profile of the hydrolysates. Overall, the porcine plasma hydrolysates were characterized by high levels of liver flavor and aftertaste, while bovine minced meat and heart hydrolysates were high in umami taste. The porcine hemoglobin hydrolysates were low in all sensory attributes, and thus very bland. All hydrolysates had a low perceived bitterness.

Considering the potential applications for each type of hydrolysate, and since both bovine meat and bovine heart hydrolysates are relatively high in umami, they would make good candidates for savory applications such as in soups or meatballs. Porcine plasma is high in liver flavor compared to the other hydrolysates, and thus, might be a good candidate for certain meat products where liver flavor is desirable, e.g., liver pâté. Since porcine hemoglobin hydrolysates proved to be the most neutral in flavor, they are a potential candidate for broader food product applications. 

### 3.2. Peptide Characteristics

#### 3.2.1. Yield

The yields of the hydrolysates are of a similar magnitude to those described by Fu et al. [7]. Yields were not determined for the glycated hydrolysates, as drying at a high temperature can lead to an excessive loss of weight due to the volatile Maillard reaction products (results not shown). In general, the yields of hydrolysates derived from bovine meat (80.0%) and porcine hemoglobin (81.4%) were relatively high, in part due to the higher protein content in these raw materials. The yields for bovine heart and porcine plasma were slightly lower at 72.7% and 73.0%, respectively.

#### 3.2.2. UV-Visible and Fluorescence Spectra of Native and Glycated Hydrolysates

**Figure 1:**
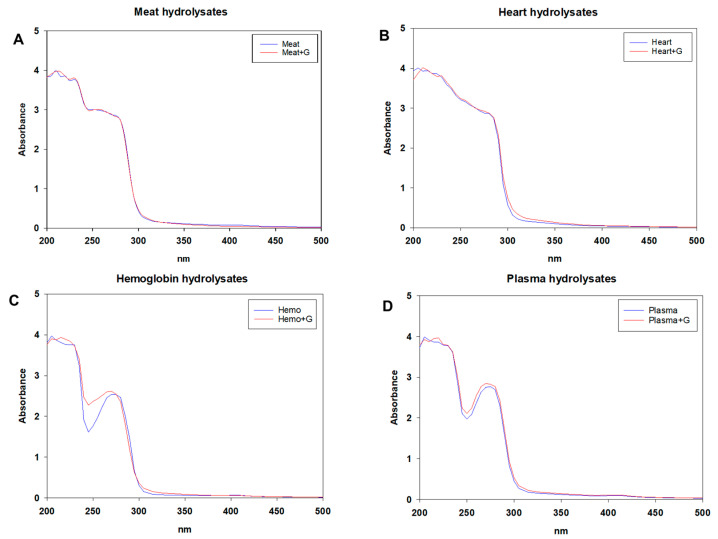
UV-scanning spectra of hydrolysates with (red) or without (blue) glucosamine (G) of (**A**) bovine meat, (**B**) bovine heart, (**C**) porcine hemoglobin, and (**D**) porcine plasma.

UV-visible spectra of native and glycated hydrolysates derived from bovine meat, bovine heart, porcine hemoglobin and porcine plasma are shown in Figure 1A–D, respectively. UV-visible analysis is a fast and easy way to monitor and characterize the progress of the Maillard reaction [22]. The formation of intermediate Maillard reaction products and late-stage melanoidin compounds during the Maillard reaction can be monitored at 320 and 420 nm, respectively [23,24]. As is especially evident for the bovine heart hydrolysates (Figure 1B), the glycated hydrolysates exhibited a slightly higher absorbance than those of native hydrolysates in the range of 300–350 nm. This fact is mainly due to the formation of intermediate Maillard reaction products.

Notably, the major peak was observed in each glycation treatment in the region of 270–280 nm (Figure 1). This fact could be ascribed to the presence of aromatic amino acids, as well as the autocondensation products of glucosamine [10,24]. There was no significant change found as a result of glucosamine addition at the wavelength of 420 nm, suggesting that no advanced glycation end-products were formed in the glycated hydrolysates.

**Figure 2:**
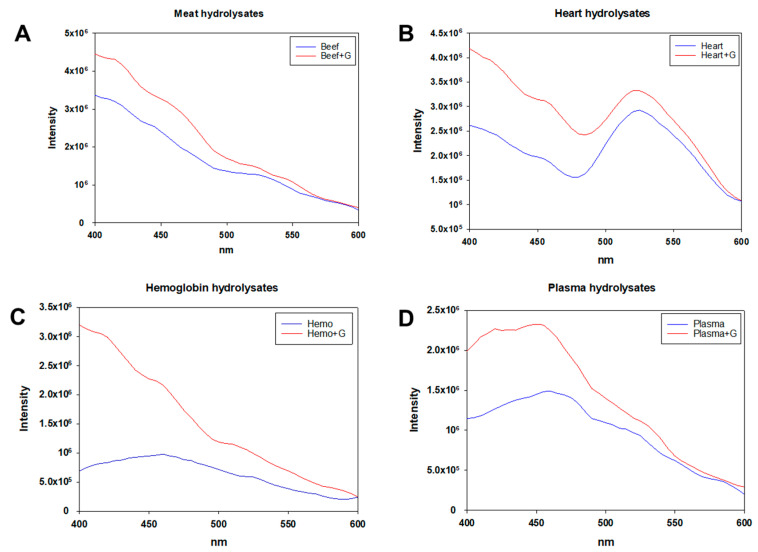
Fluorescence spectra of hydrolysates with (red) or without (blue) glucosamine (G) of (**A**) bovine meat, (**B**) bovine heart, (**C**) porcine hemoglobin, and (**D**) porcine plasma.

Fluorescence spectroscopy can serve as a method of detecting the fluorescent products generated by the Maillard reaction [25]. The fluorescent products can be detected at the excitation wavelength of 347 nm, as well as the emission wavelength of 400–600 nm [10,26]. Fluorescence spectra of native hydrolysates, glycated hydrolysates derived from bovine meat, bovine heart, porcine hemoglobin, and porcine plasma are illustrated in Figure 2A–D, respectively. Apart from bovine heart, the remaining samples tended to display a progressive downtrend of fluorescence intensity with the emission wavelength ranging from 400 nm to 600 nm (Figure 2A,C,D). By contrast, bovine heart samples showed a downward trend in the region of 400–480 nm, followed by a major peak around the region of 500–600 nm (Figure 2B). It has previously been demonstrated that several fluorescent products contribute to the increased fluorescence intensity by glycation using glucosamine, such as pentosidine, argpyrimidine, pentodilysine, pirropyridine, as well as the autocondensation products of glucosamine, even at a moderate reaction temperature [10].

#### 3.2.3. Peptide Size Distribution of Native and Glycated Hydrolysates

**Figure 3:**
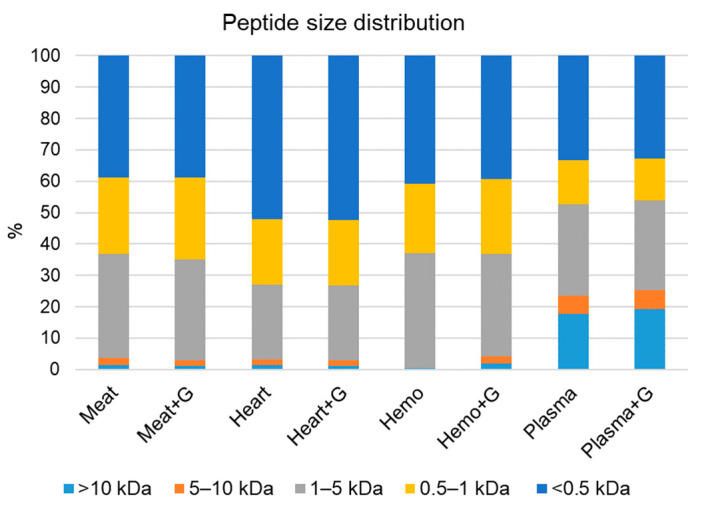
Peptide size distribution of native and glycated hydrolysates.

The peptide size distributions of native hydrolysates, glycated hydrolysates derived from bovine meat, bovine heart, porcine hemoglobin, and porcine plasma were determined and displayed in Figure 3. Plasma samples tended to show higher percentages of high MW peptide fraction (>5 kDa) than those derived from other raw materials (Figure 3), as also seen in our previous study [7]. This phenomenon might be due to the high resistance of plasma proteins towards Protease A, or to its tendency to aggregate during high-temperature heat treatment [7]. By contrast, heart hydrolysates contained the highest proportion of low MW peptide fraction (<1 kDa) (Figure 3) among the hydrolysates, suggesting that heart muscle proteins are easily degraded into small peptides or free amino acids. Generally, there was no significant change in the peptide size distribution between native and glycated hydrolysates, which is not surprising, considering the molecular weight of glucosamine is 161 Da. Furthermore, this is in line with the results of both the sensory and the volatile analysis (discussed below)—indicating that the extent of glycation was relatively low.

### 3.3. Volatile Profiles Determined by GC-MS

In total, 69 volatiles were identified including aldehydes (20), alcohols (12), and ketones (10), in addition to typical Maillard reaction compounds such as furans and pyrazines (see Supplementary Table S2). The chromatograms are shown in Supplementary Figure S1.

For 13 of the 69 detected volatiles (Table S2), there was a statistically significant effect of glucosamine addition. The relative concentrations of the 13 compounds are shown in Table 3.

The different effects of glucosamine addition were observed for bovine meat, bovine heart, porcine hemoglobin, and porcine plasma hydrolysates, indicating that the effect of the addition of glucosamine on volatile compound development in animal hydrolysates is rather raw material specific. The lipid oxidation product 3-methyl-2-butenal was the only compound in which an effect of glucosamine was observed for more than one raw material (bovine meat and porcine hemoglobin).

Yolk flavor, the only flavor attribute that increased with the addition of glucosamine (Table 2), is often associated with the presence of sulfur compounds such as sulfides and thiazoles [28], however, many aldehydes also act as odor-active compounds within an egg yolk [29]. These compounds originate from either the autoxidation of unsaturated fatty acids, thermal oxidation of saturated triacylglycerols, or Strecker degradation [30]. Both 2-methylbutanal and 3-methylbutanal (Table 3) have been detected in egg yolk, the latter being notable for significant odor activity [28]. In the present experiment, a difference in 3-methylbutanal and 2-methylbutanal content between hydrolysates with and without glucosamine was only found for porcine plasma, yet in those cases, this actually represented a decrease with the addition of glucosamine (Table 3). To our knowledge, out of the remaining volatiles identified via GC-MS where a difference was detected between hydrolysates with and without glucosamine, only D-limonene has previously been identified in egg yolk, however it does not appear to be one of the main odor-active compounds [29]. The presence of the terpenes D-limonene and myrcene in the hydrolysates is likely due to the animal feed.

Apparently, there was no correlation between the results of the sensory analysis and the volatile compound analysis, indicating that the volatile compounds from Table 3 were not necessarily very odor-active. These findings are in line with the findings of the peptide characteristics analyses—all in all, showing that the extent of the Maillard reaction was relatively low under the current experimental conditions.

Further investigation into the effect of heated systems on the flavor-forming potential of hydrolysates both with and without added glucosamine is needed, as application of the hydrolysates at higher temperatures, e.g., during cooking, would be expected to lead to more extensive Maillard reactions than witnessed here.

## 4. Conclusions

In summary, glucosamine addition at a ratio of 1:10 relative to the protein content of the raw material (bovine minced meat and heart, porcine hemoglobin and plasma) provided only minor changes to sensory, volatile, and peptide-characteristics of animal hydrolysates under the conditions investigated, including a slightly increased perception of sweet taste, umami taste, and yolk flavor.

Due to the low perceived bitterness of all native and glycated hydrolysates there is definitely potential for application in food products. As raw material heavily influences the sensory attributes of the hydrolysate, this will influence the specific application of each hydrolysate.

Glucosamine-induced Maillard reaction can have an effect on the flavor and peptide characteristics of hydrolysates, however the effect observed here was minor, indicating the need for a more extensive Maillard reaction in order to fully take advantage of the glycation process.

## Figures and Tables

**Table 1:** List of sensory attributes with definitions developed for the profiling.

Sensory Attribute ^1^	Scale	Definition with Reference Materials ^2^
**Odor**		**Odor associated with**
Animal-O	‘not at all’ → ‘very much’	Animal note reminiscent of Parma ham/Prosciutto di Parma
Raw meat-O	‘not at all’ → ‘very much’	Odor characteristic reminiscent of fresh minced beef (4–7% fat)
**Taste**		
Umami-T	‘not at all’ → ‘very much’	Taste sensation of monosodium glutamate (0.7 g/L water)
Sweet-T	‘not at all’ → ‘very much’	Taste sensation of sucrose (12 g/L water)
Salt-T	‘not at all’ → ‘very much’	Taste sensation of sodium chloride (2.0 g/L water)
Bitter-T	‘not at all’ → ‘very much’	Taste sensation of caffeine (0.58 g/L water)
**Flavor**		**Aromatic taste sensation associated with**
Metallic-F	‘not at all’ → ‘very much’	Metallic sensation reminiscent of ferrous sulfate (0.016 g/L water)
Liver-F	‘not at all’ → ‘very much’	Flavor sensation reminiscent of liver pâté
Yolk-F	‘not at all’ → ‘very much’	Flavor sensation reminiscent of pasteurized egg yolk
Fish sauce-F	‘not at all’ → ‘very much’	Flavor sensation reminiscent of Asian fish sauce
Sulfur-F	‘not at all’ → ‘very much’	Sulfury flavor note reminiscent of boiled egg yolk
Toasted Burnt-F	‘not at all’ → ‘very much’	Toast
**Aftertaste (AT)**		**Aftertaste associated with**
Overall Intensity-AT	‘not at all’ → ‘very much’	Overall intensity
Umami-AT	‘not at all’ → ‘very much’	Taste sensation of monosodium glutamate (0.7 g/L water)
Bitter-AT	‘not at all’ → ‘very much’	Taste sensation of caffeine (0.58 g/L water)
Metallic-AT	‘not at all’ → ‘very much’	Metallic taste sensation reminiscent of ferrous sulfate (0.016 g/L water)
Liver-AT	‘not at all’ → ‘very much’	Flavor sensation reminiscent of liver pâté

^1^ Suffix to sensory attributes indicates method of assessment by panelists: -O, odor, -T, taste, -F, flavor and -AT, aftertaste. ^2^ Definitions of sensory attributes as agreed on between the panelists during vocabulary development.

**Table 2:** Panel mean intensity scores of the sensory attributes for the hydrolysates rated on a 15-cm unstructured line scale, with *p*-values and suffixes (a, b, c, d, e) annotating sample differences. G indicates glucosamine addition.

Sensory Attributes ^1^	Meat	Meat + G	Heart	Heart + G	Hemo	Hemo + G	Plasma	Plasma + G	*p*-Value ^2^
**Odor (O)**									
Animal-O	6.9 b	7.4 b	8.4 b	8.0 b	1.8 a	3.8 a	11.4 c	12.2 c	<0.001
Raw meat-O	9.2 bc	9.7 c	9.8 c	9.4 bc	2.4 a	5.2 ab	5.4 ab	5.3 ab	<0.001
**Taste (T)**									
Umami-T	10.1 ab	11.0 ab	11.6 b	12.1 b	7.7 a	8.9 ab	7.9 a	8.9 ab	<0.001
Sweet-T	4.6 b	4.9 b	4.5 b	5.1 b	2.1 a	3.5 ab	3.8 ab	4.8 b	<0.001
Salt-T	3.1 ab	4.0 ac	3.8 ac	3.8 ac	2.5 a	3.2 ab	6.1 bc	6.6 c	0.001
Bitter-T	5.1 a	5.9 a	5.8 a	4.5 a	3.3 a	4.3 a	6.1 a	6.1 a	0.031
**Flavor (F)**									
Metallic-F	7.8 ac	8.6 bc	9.3 c	8.1 ac	5.9 a	6.4 ab	8.7 bc	9.8 c	<0.001
Liver-F	4.4 a	4.7 a	6.1 ab	4.9 a	4.1 a	4.3 a	10.4 bc	11.2 c	<0.001
Yolk-F	8.9 bc	9.7 bc	10.2 c	10.3 c	4.4 a	7.6 ac	5.9 ab	6.2 ab	<0.001
Fish sauce-F	2.6 ab	4.1 b	3.7 ab	3.4 ab	1.7 a	2.5 ab	3.8 ab	4.1 b	0.011
Sulfur-F	4.9 ab	5.0 b	5.4 b	5.3 b	2.3 a	3.9 ab	4.1 ab	4.2 ab	0.014
Toasted Burnt-F	1.2 a	1.5 ab	1.2 a	1.4 ab	0.8 a	1.3 ab	3.9 bc	4.1 cb	<0.001
**After taste (AT)**									
Overall Intensity-AT	8.5 bc	9.1 cd	9.6 ce	9.3 ce	5.7 a	6.4 ab	11.0 de	11.4 e	<0.001
Umami-AT	8.8 ac	9.4 bc	10.2 c	10.0 bc	5.7 a	7.1 ac	6.7 ab	6.7 ab	<0.001
Bitter-AT	5.3 ab	6.6 b	6.3 ab	5.5 ab	3.7 a	3.7 a	6.3 ab	6.2 ab	0.002
Metallic-AT	5.8 ac	6.8 bcd	7.2 cd	6.5 bcd	4.1 a	5.1 ab	7.6 cd	8.3 d	<0.001
Liver-AT	3.0 a	3.6 a	4.4 a	3.4 a	2.3 a	2.4 a	9.8 b	10.7 b	<0.001

^1^ Suffix to sensory terms indicates method of assessment by panelists: -O, odor, -T, taste, -F, flavor, -AT, aftertaste. ^2^ Significance level: Different letters (a, b, c, d, e) within the same rows indicate significant (*p* < 0.05) differences between samples according to Tukey’s HSD test.

**Table 3:** Volatile compounds where glucosamine (G) addition had an effect on quantities present in the hydrolysates. Relative quantity in hydrolysate without glucosamine is set to 1, and hydrolysate + G is indicated as fold increase.

Volatile	Odor ^1^	Meat + G	Heart + G	Hemo + G	Plasma + G	Retention Index ^3^		
						Exp.	Auth. std.	Literature
2-Ethyl-1-hexanol	Rose, green		1.8			1500	1503	
2-Methylbutanal	Cocoa, almond				0.5	911		880–963
3-Methylbutanal	Malt				0.6	915	917	
(*E*)-2-Butenal	Flower		1.9			1030		1002–1084
(*E*)-2-Methyl-2-butenal	Green, fruit	4.5				1088		1012–1113
3-Methyl-2-butenal	Almond, roasted ^2^	6.4		4.6		1197		1189–1236
5-Ethylcyclopent-1-enecarboxaldehyde	---		0.7			1423		1399–1428
4-Ethylbenzaldehyde	Sweet	1.9				1722		1711–1753
Estragole	Licorice, anise	2.3				1681		1624–1701
Myrcene	Balsamic, must, spice		4.4			1172	1170	
D-Limonene	Citrus		6.3			1194	1197	
1-Methyl-4-(1-methylethylidene)-cyclohexene	Pine, plastic		10.8			1291		1233–1323
Benzonitrile	Rancid	2.3				1614		1570–1637

^1^ Odor descriptors are from www.flavornet.org (accessed on 16 June 2020). ^2^ Odor descriptors are from [27]. ^3^ Exp. = experimental value, Auth. std. = authentic standard.

## Data Availability

The data presented in the study are available from the corresponding author upon request.

## Supplementary Materials

The following are available online at https://www.mdpi.com/article/10.3390/foods10123008/s1, Table S1: Hydrolysates produced by 5 h enzymatic hydrolysis with Protease A based on different types of raw material and glucosamine (G) addition at a ratio of 1:10 relative to the protein content of the raw materials. Table S2: Peak areas (×10^−5^) of the volatile compounds in the hydrolysates with or without glucosamine (G) relative to the peak area of the internal standard. Different letters within the same rows indicate significant (*p* < 0.05) differences according to Tukey’s test. Figure S1: Chromatograms showing the volatile composition of hydrolysates of **A** minced bovine meat (BEEF), **B** bovine heart (HEART), **C** porcine hemoglobin (HEMO), and **D** porcine plasma (PLASMA) in dublicates (A and B) with or without glucosamine (G).
