# Supplementary material for: Flavor Characterization of Animal Hydrolysates and Potential of Glucosamine in Flavor Modulation
Source: Foods. 2021 Dec 4;10(12):3008. doi: 10.3390/foods10123008 (PMC8701079; doi:10.3390/foods10123008)
Supplement: Supplementary file 1 [file foods-10-03008-s001.zip › foods-1450236-supplementary.pdf]

Table S1. Hydrolysates produced by 5 h enzymatic hydrolysis with Protease A based on different types of raw material and glucosamine (G) addition at a ratio of 1:10 relative to the protein content of the raw materials.

Animal source	Glucosamine addition	Inactivation temp.	Product name
Bovine meat	No	90	Meat
	G 1:10	90	Meat+G
Bovine heart	No	90	Heart
	G 1:10	90	Heart+G
Porcine hemoglobin	No	90	Hemo
	G 1:10	90	Hemo+G
Porcine plasma	No	85	Plasma
	G 1:10	85	Plasma+G

Table S2. Peak areas (x10^{-5}) of the volatile compounds in the hydrolysates with or without glucosamine (G) relative to the peak area of the internal standard. Different letters within the same rows indicate significant (P<0.05) differences according to Tukey's test.

Compound	Meat	Meat+G	Heart	Heart+G	Hemo	Hemo+G	Plasma	Plasma+G	Retention index		
									Exp.	Auth. std.	Literature
**Alcohol**											
Ethanol	287ab	411a	180ab	176ab	189ab	271ab	82b	115ab	938		883-972
1-Butanol	342ab	425ab	409ab	559a	67c	184bc	83c	60c	1163	1165	
2-Methyl-1-butanol	28b	143b	384a	481a	111b	88b	125b	113b	1220		1158-1244
3-Methyl-1-butanol	142e	258de	658ab	821a	241de	182de	519bc	403cd	1222	1238	
3-Methyl-3-buten-1-ol	158b	157b	264b	306b	146b	155b	598a	500a	1265		1221-1277
1-Pentanol	13795a	12596a	1646b	1479b	355b	5629ab	688b	593b	1271	1274	
1-Hexanol	2929ab	3980a	609c	663c	377c	1315bc	193c	203c	1369	1372	
1-Octen-3-ol	12660ab	16047a	11936ab	6687ab	1600b	7394ab	7136ab	9715ab	1461	1464	

1-Heptanol	2279a	2198ab	455abc	476abc	73c	1005abc	186bc	196bc	1467	1470	
2-Ethyl-1-hexanol	377bc	785bc	**1052b**	**1880a**	188c	463bc	180c	174c	1500	1503	
1-Octanol	1689ab	1843a	622ab	765ab	452b	994ab	1401ab	1746a	1569	1570	
(*E*)-2-Octen-1-ol	2360bc	2762bc	1716bc	1366bc	978c	1642bc	3549ab	5835a	1627		1577-1649
**Aldehyde**											
2-Methylpropanal	259c	927abc	1083ab	1407a	615abc	410bc	1187ab	643abc	812	813	
2-Propenal	120bc	822abc	961ab	1287a	105bc	182bc	67c	86bc	840		828-876
2-Methylbutanal	2241de	6189cd	11117b	9533bc	419e	1457e	**19984a**	**10176b**	911		880-963
3-Methylbutanal	6642e	19183bcde	30848bc	27629bcd	15876cde	12399de	**58481a**	**33694b**	915	917	
Pentanal	767bc	3196ab	5408a	3921a	167c	739bc	349c	246c	975	983	
(*E*)-2-Butenal	34c	33c	**258b**	**499a**	8c	13c	33c	21c	1030		1002-1084
Hexanal	6238b	23054b	95480a	59608ab	631b	5693b	705b	758b	1081	1082	
(*E*)-2-Methyl-2-butenal	**730b**	**3312a**	2904a	2536a	ND	668b	86b	198b	1088		1012-1113
Heptanal	1395bcd	4521ab	5199a	4180abc	267d	908cd	409d	347d	1188	1189	
3-Methyl-2-butenal	**513cd**	**3304a**	2831ab	2471b	**225d**	**1036c**	80d	181d	1197		1189-1236
Octanal	1040abc	2840a	2889a	2302ab	404c	883bc	541bc	511bc	1302	1306	
Nonanal	2514ab	6374ab	7183a	6883a	1307b	2097ab	1299b	1276b	1399	1402	
5-Ethylcyclopent-1-enecarboxaldehyde	52c	239c	**946a**	**624b**	16c	51c	27c	28c	1423		1399-1428
(*E*)-2-Octenal	270bc	1271b	4158a	3972a	77c	279bc	188c	293bc	1437	1444	
Decanal	534	988	1103	1732	222	488	453	340	1507	1511	
Benzaldehyde	6726ab	21263a	8849ab	10564ab	18070ab	16900ab	4186b	6643ab	1531	1531	
(*E*)-2-Nonenal	185b	668b	1861a	2855a	ND	119b	51b	42b	1546	1551	
Benzeneacetaldehyde	234c	916bc	2077a	1819ab	797bc	676c	1880ab	1135abc	1651	1659	

4-Ethylbenzaldehyde	**256b**	**481a**	72bc	87bc	5c	102bc	12c	7c	1722		1711-1753
3,5-Dimethylbenzaldehyde	604	1063	1443	870	977	1176	785	702	1831		1837
**Alkane**											
Heptane	162	190	234	148	29	96	46	33	699		700
Octane	310	500	566	394	129	231	187	140	800		800
2,2,4,6,6-Pentamethylheptane	442	169	377	271	161	83	491	387	943		915-957
Tridecane	52	145	105	359	11	20	21	13	1309		1300
Pentadecane	64	193	190	494	11	29	26	17	1499		1500
**Carboxylic acid**											
Acetic acid	182	171	236	287	60	317	153	99	1456		1400-1536
**Ester**											
Ethyl Acetate	177	328	109	278	223	260	427	271	891		850-921
**Furan**											
2-Pentylfuran	58b	144ab	920a	356ab	12b	41b	31b	29b	1240		1193-1265
Butyrolactone	246	211	216	121	18	126	58	38	1640		1592-1673
**Ketone**											
Acetone	1476	1725	2228	1557	1762	1228	1735	2622	815		775-854
2-Butanone	5117abc	10435a	6420abc	8712ab	938c	2639bc	3069bc	2563bc	903	906	
3-Methyl-2-butanone	265	622	623	604	954	661	591	434	926		918-989
2-Pentanone	585c	619c	7929ab	6242b	1565c	1095c	10676a	6926ab	974	977	
2,3-Butanedione	3529b	7492ab	13060a	13233a	249b	1616b	303b	159b	980	985	
2-Heptanone	1326ab	1270ab	2143a	1570ab	278b	673ab	1945a	1778ab	1187	1189	

Acetoin	8094bc	17092abc	31950a	25364ab	3c	6636bc	10c	12c	1295	1307	
6-Methyl-5-hepten-2-one	422ab	643a	534ab	616a	117c	306bc	190c	169c	1350	1353	
2-Nonanone	64bc	84bc	218a	139ab	18c	36bc	192a	216a	1396	1398	
2-Decanone	56c	88bc	110abc	112abc	19c	28c	323ab	371a	1502		1463-1519
**Phenol**											
Estragole	**23b**	**52a**	32ab	19b	24b	23b	11b	12b	1681		1624-1701
Phenol	178	289	264	255	165	149	158	197	2014		1946-2045
p-Cresol	29de	37cde	85bc	70cd	11e	19e	129ab	164a	2093		2031-2126
2,4-Bis(1,1-dimethylethyl)phenol	42	76	17	79	48	78	22	48	2296		2270-2330
**Pyrazine**											
Pyrazine	42bc	105ab	100ab	141a	ND	50bc	46bc	117ab	1212		1179-1257
**Terpene**											
Myrcene	71b	220ab	**107b**	**465a**	5b	60b	18b	17b	1172	1170	
D-Limonene	157bc	528b	**178bc**	**1123a**	8c	93bc	36c	37c	1194	1197	
1-Methyl-4-(1-methylethylidene)-cyclohexene	44bc	117b	**35bc**	**381a**	ND	22c	11c	12c	1291		1233-1323
α-Terpeniol	154	269	80	257	6	179	22	19	1710	1716	
**Other**											
Acetonitrile	455	490	256	233	123	424	25	146	1002		988-1045
Toluene	288	319	384	234	333	479	352	266	1029		1011-1093
(1-	435	139	253	442	332	144	384	303	1174		1150-1236

Methylethyl)benzene										
Styrene	192	231	185	112	57	222	86	115	1263	1229-1310
α-Methylstyrene	421	336	487	680	796	332	658	700	1341	1295-1366
Benzonitrile	**69b**	**160a**	92b	85b	67b	92b	42b	40b	1614	1570-1637
Dimethyl sulfone	67	42	186	31	19	30	21	28	1916	1869-1914
1,3-Di-isopropylnaphthalene	46	134	24	210	4	24	5	5	2164	2139-2162
1,7-Di-isopropylnaphthalene	35	101	8	160	2	18	3	1	2179	2154-2178

A

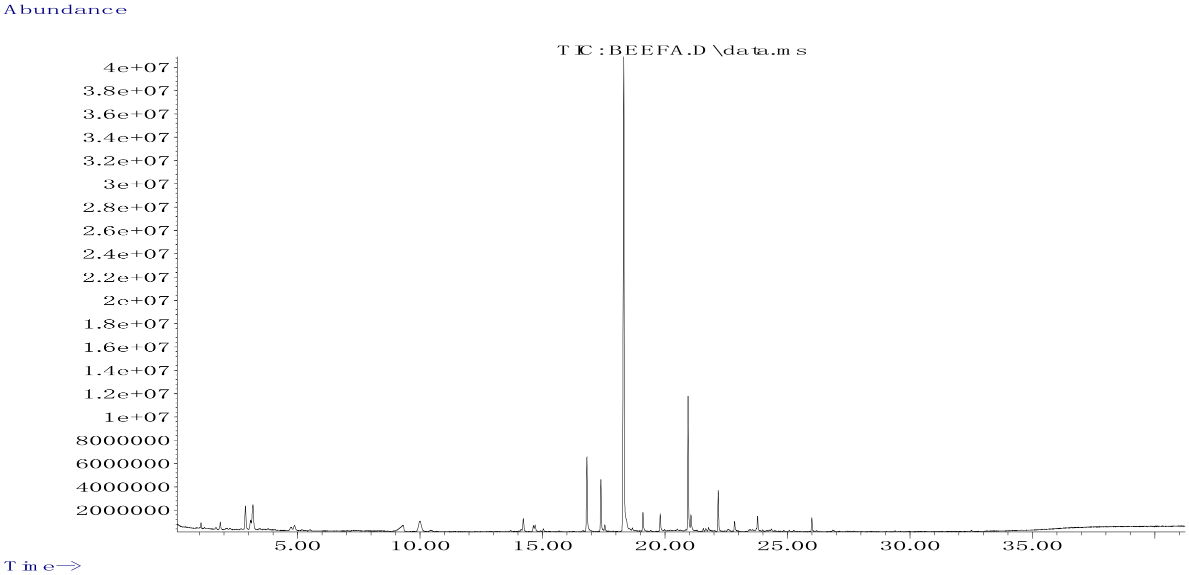
Abundance
TIC: BEEFA.D\data.ms
4e+07
3.8e+07
3.6e+07
3.4e+07
3.2e+07
3e+07
2.8e+07
2.6e+07
2.4e+07
2.2e+07
2e+07
1.8e+07
1.6e+07
1.4e+07
1.2e+07
1e+07
8000000
6000000
4000000
2000000
5.00
10.00
15.00
20.00
25.00
30.00
35.00
Time-->

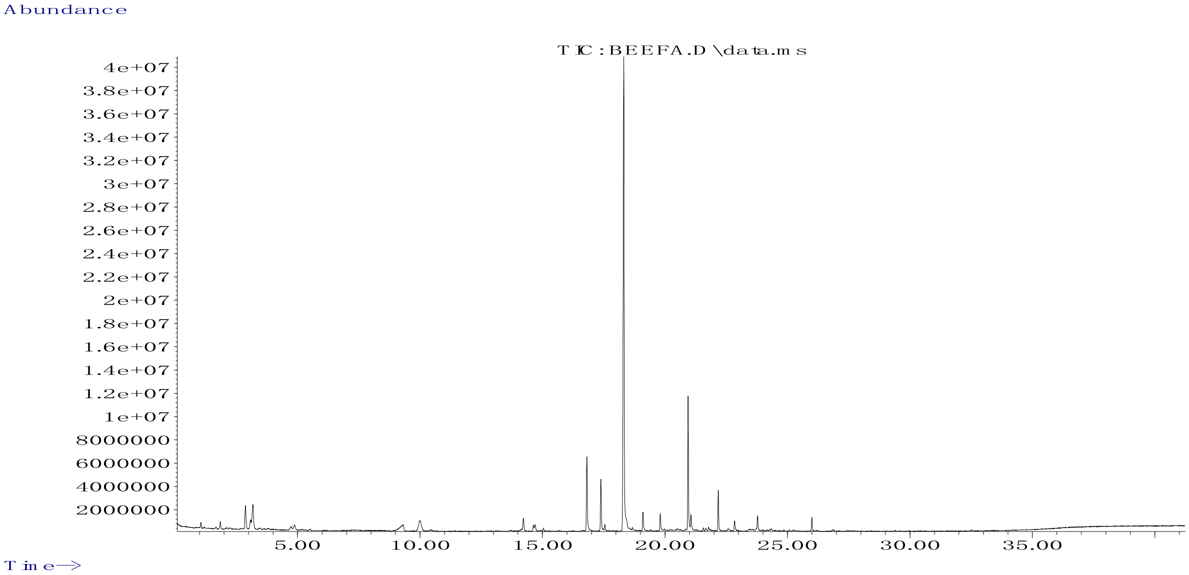
Abundance
TIC: BEEFA.D\data.ms
4e+07
3.8e+07
3.6e+07
3.4e+07
3.2e+07
3e+07
2.8e+07
2.6e+07
2.4e+07
2.2e+07
2e+07
1.8e+07
1.6e+07
1.4e+07
1.2e+07
1e+07
8000000
6000000
4000000
2000000
5.00
10.00
15.00
20.00
25.00
30.00
35.00
Time-->

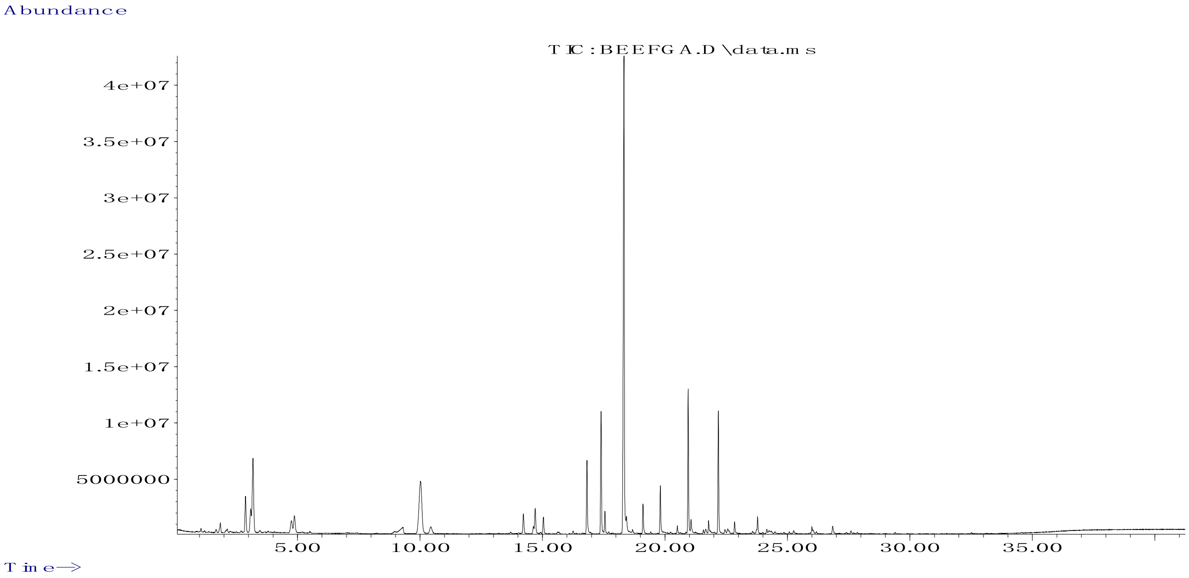
Abundance
TIC: BEEFGA.D\data.ms
4e+07
3.5e+07
3e+07
2.5e+07
2e+07
1.5e+07
1e+07
5000000
5.00
10.00
15.00
20.00
25.00
30.00
35.00
Time-->

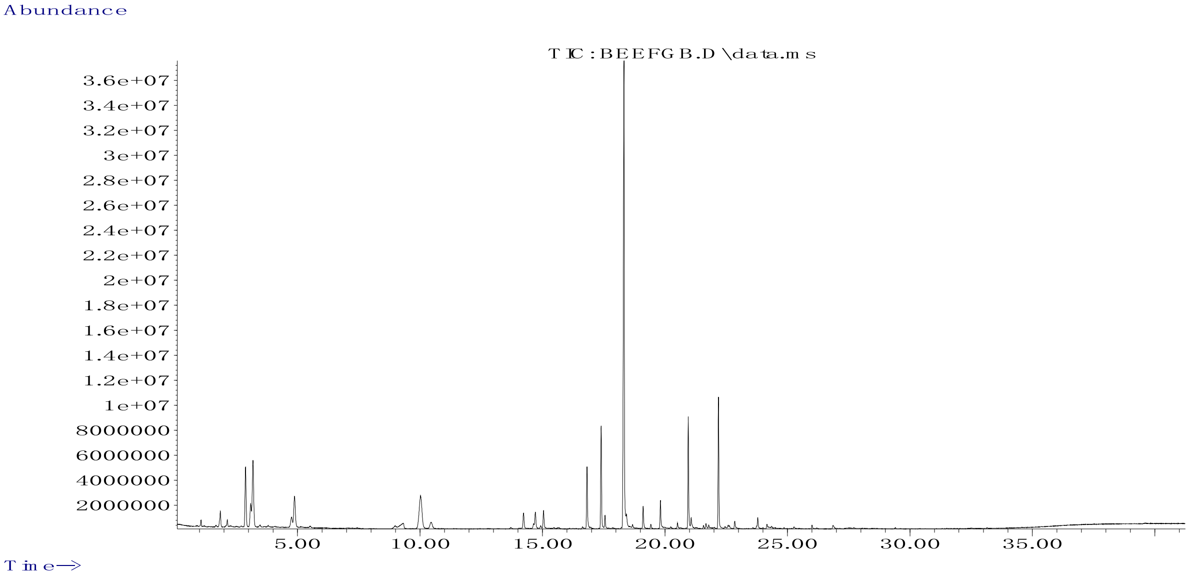
Abundance
TIC: BEEFGB.D\data.ms
3.6e+07
3.4e+07
3.2e+07
3e+07
2.8e+07
2.6e+07
2.4e+07
2.2e+07
2e+07
1.8e+07
1.6e+07
1.4e+07
1.2e+07
1e+07
8000000
6000000
4000000
2000000
5.00
10.00
15.00
20.00
25.00
30.00
35.00
Time-->

B

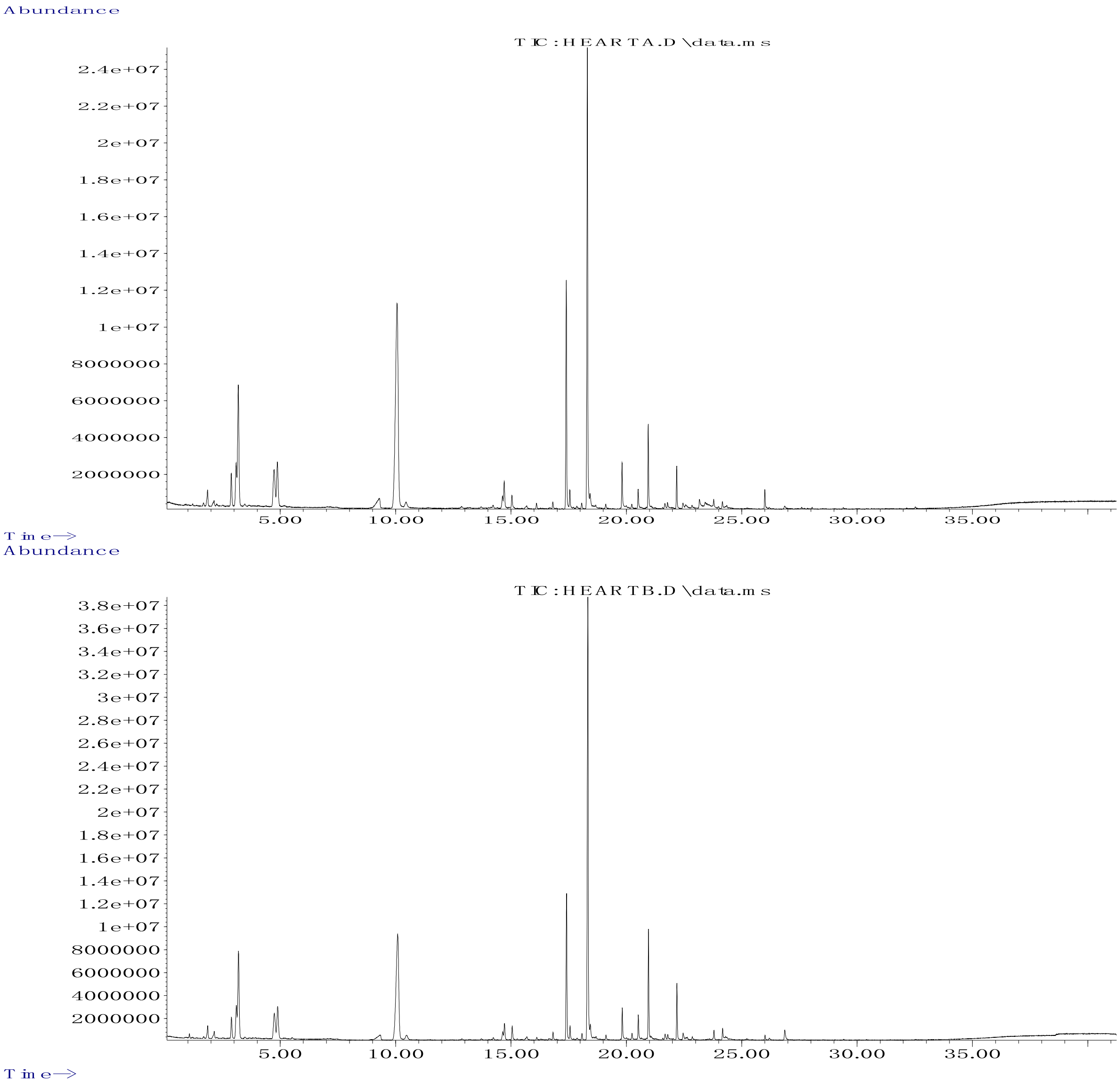
Abundance
TIC: HEARTA.D\data.ms
2.4e+07
2.2e+07
2e+07
1.8e+07
1.6e+07
1.4e+07
1.2e+07
1e+07
8000000
6000000
4000000
2000000
5.00
10.00
15.00
20.00
25.00
30.00
35.00
Time-->
Abundance
TIC: HEARTB.D\data.ms
3.8e+07
3.6e+07
3.4e+07
3.2e+07
3e+07
2.8e+07
2.6e+07
2.4e+07
2.2e+07
2e+07
1.8e+07
1.6e+07
1.4e+07
1.2e+07
1e+07
8000000
6000000
4000000
2000000
5.00
10.00
15.00
20.00
25.00
30.00
35.00
Time-->

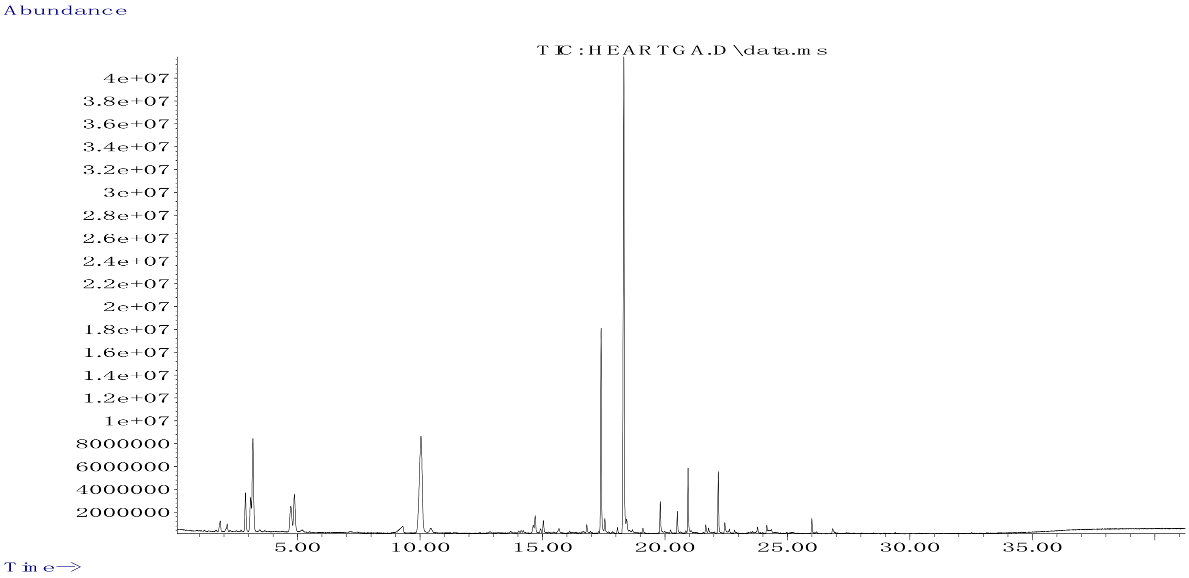
Abundance
TIC: HEARTGA.D\data.ms
4e+07
3.8e+07
3.6e+07
3.4e+07
3.2e+07
3e+07
2.8e+07
2.6e+07
2.4e+07
2.2e+07
2e+07
1.8e+07
1.6e+07
1.4e+07
1.2e+07
1e+07
8000000
6000000
4000000
2000000
5.00
10.00
15.00
20.00
25.00
30.00
35.00
Time-->

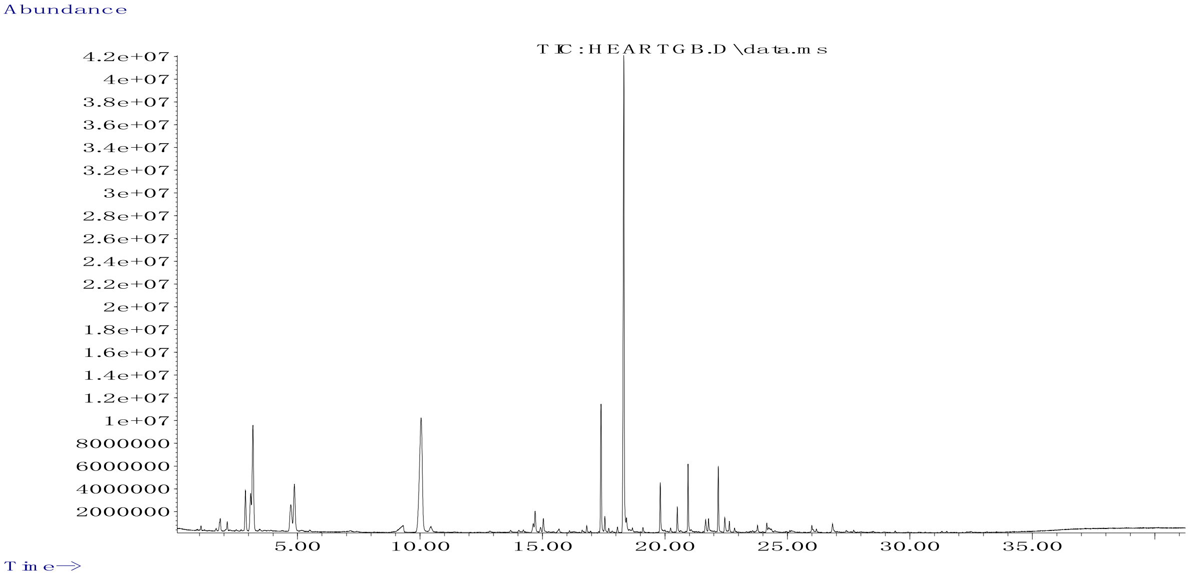
Abundance
TIC: HEARTGB.D\data.ms
4.2e+07
4e+07
3.8e+07
3.6e+07
3.4e+07
3.2e+07
3e+07
2.8e+07
2.6e+07
2.4e+07
2.2e+07
2e+07
1.8e+07
1.6e+07
1.4e+07
1.2e+07
1e+07
8000000
6000000
4000000
2000000
5.00
10.00
15.00
20.00
25.00
30.00
35.00
Time-->

C

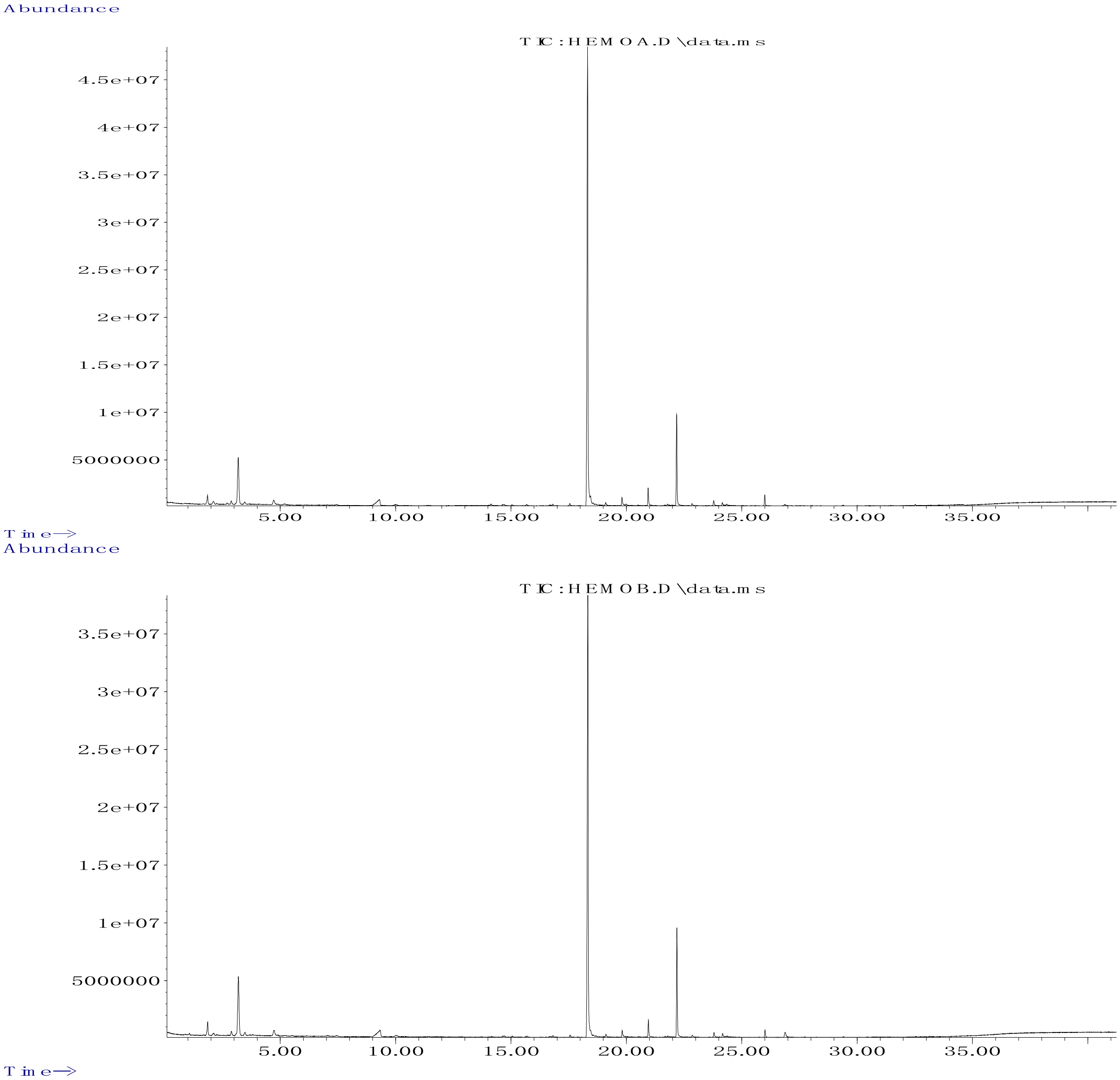

Abundance
TIC: HEMOA.D\data.ms
4.5e+07
4e+07
3.5e+07
3e+07
2.5e+07
2e+07
1.5e+07
1e+07
5000000
5.00
10.00
15.00
20.00
25.00
30.00
35.00
Time-->
Abundance
TIC: HEMOB.D\data.ms
3.5e+07
3e+07
2.5e+07
2e+07
1.5e+07
1e+07
5000000
5.00
10.00
15.00
20.00
25.00
30.00
35.00
Time-->

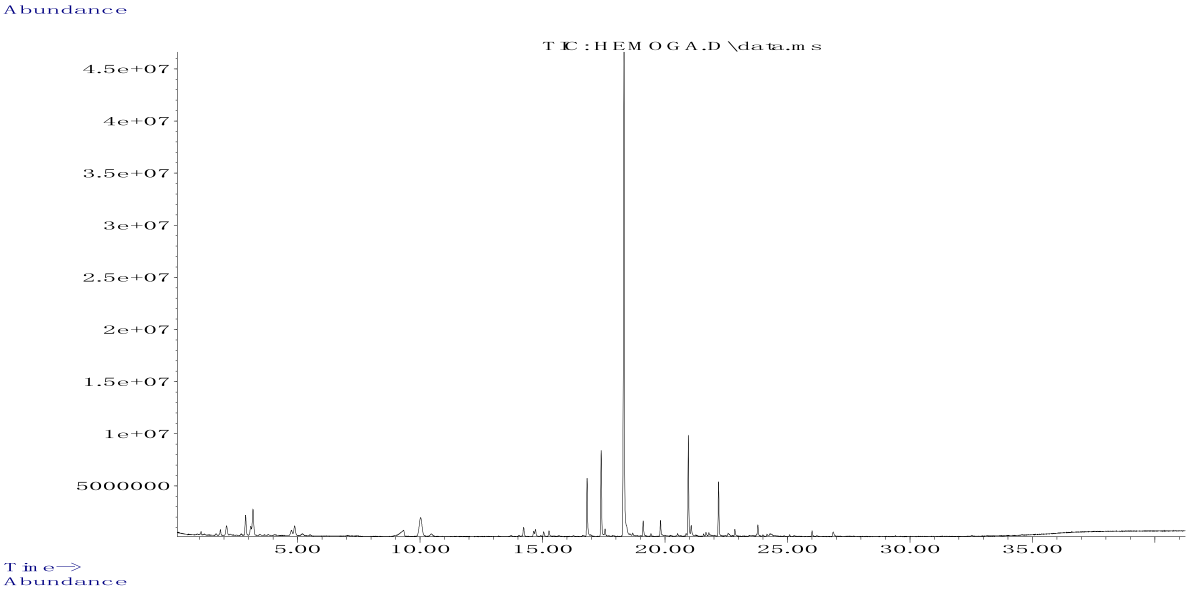
Abundance
TIC: HEMOGA.D\data.ms
4.5e+07
4e+07
3.5e+07
3e+07
2.5e+07
2e+07
1.5e+07
1e+07
5000000
5.00
10.00
15.00
20.00
25.00
30.00
35.00
Time-->

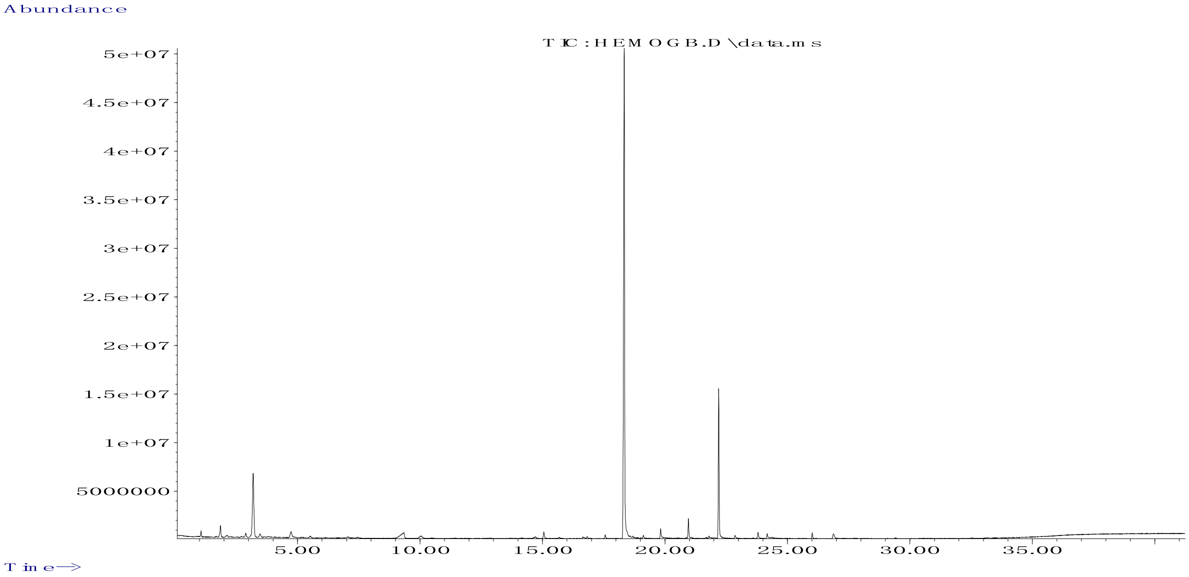
Abundance
TIC: HEMOGB.D\data.ms
5e+07
4.5e+07
4e+07
3.5e+07
3e+07
2.5e+07
2e+07
1.5e+07
1e+07
5000000
5.00
10.00
15.00
20.00
25.00
30.00
35.00
Time-->

D

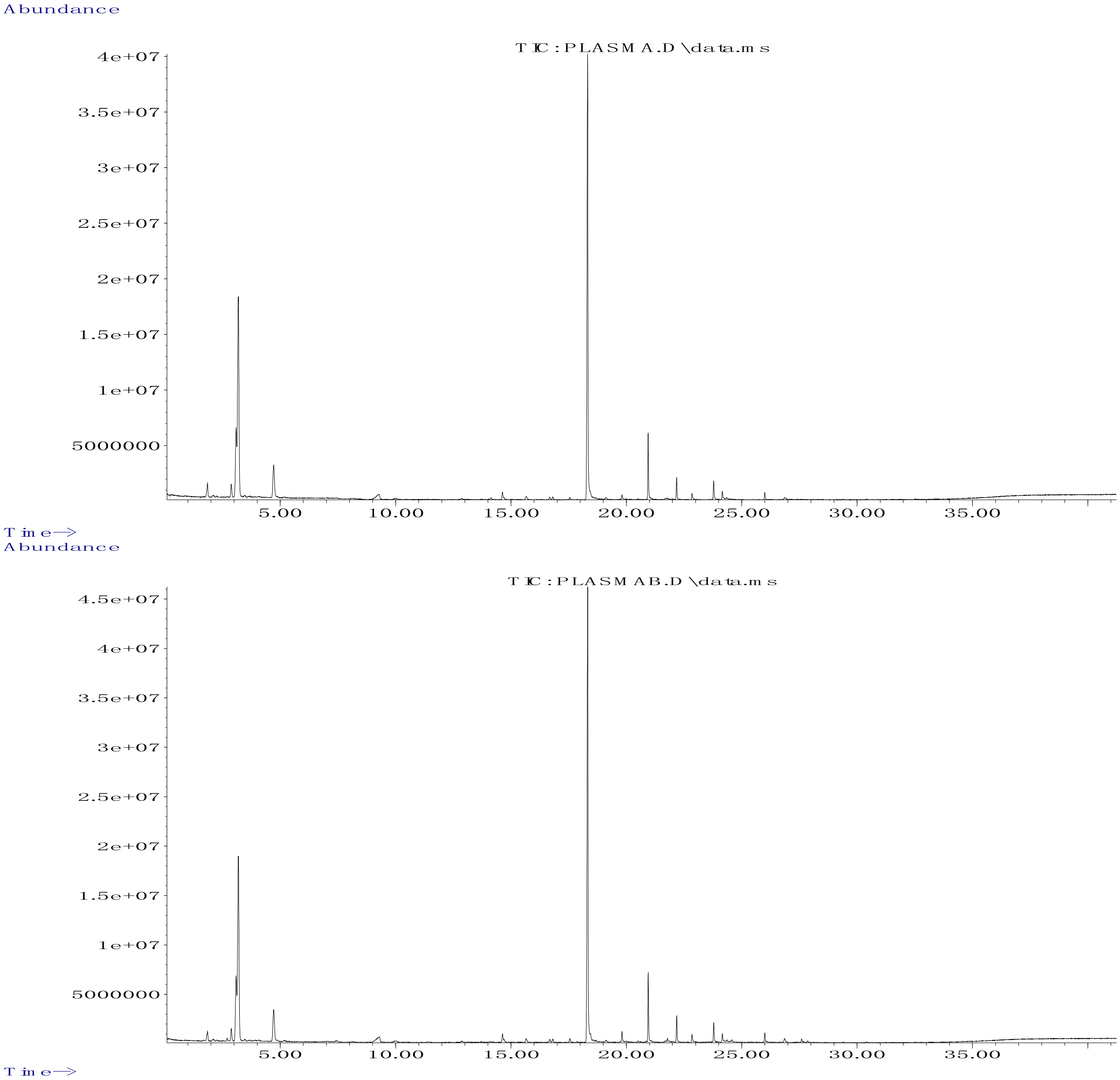

Abundance
TIC: PLASMA.D\data.ms
4e+07
3.5e+07
3e+07
2.5e+07
2e+07
1.5e+07
1e+07
5000000
5.00
10.00
15.00
20.00
25.00
30.00
35.00
Time-->
Abundance
TIC: PLASMAB.D\data.ms
4.5e+07
4e+07
3.5e+07
3e+07
2.5e+07
2e+07
1.5e+07
1e+07
5000000
5.00
10.00
15.00
20.00
25.00
30.00
35.00
Time-->

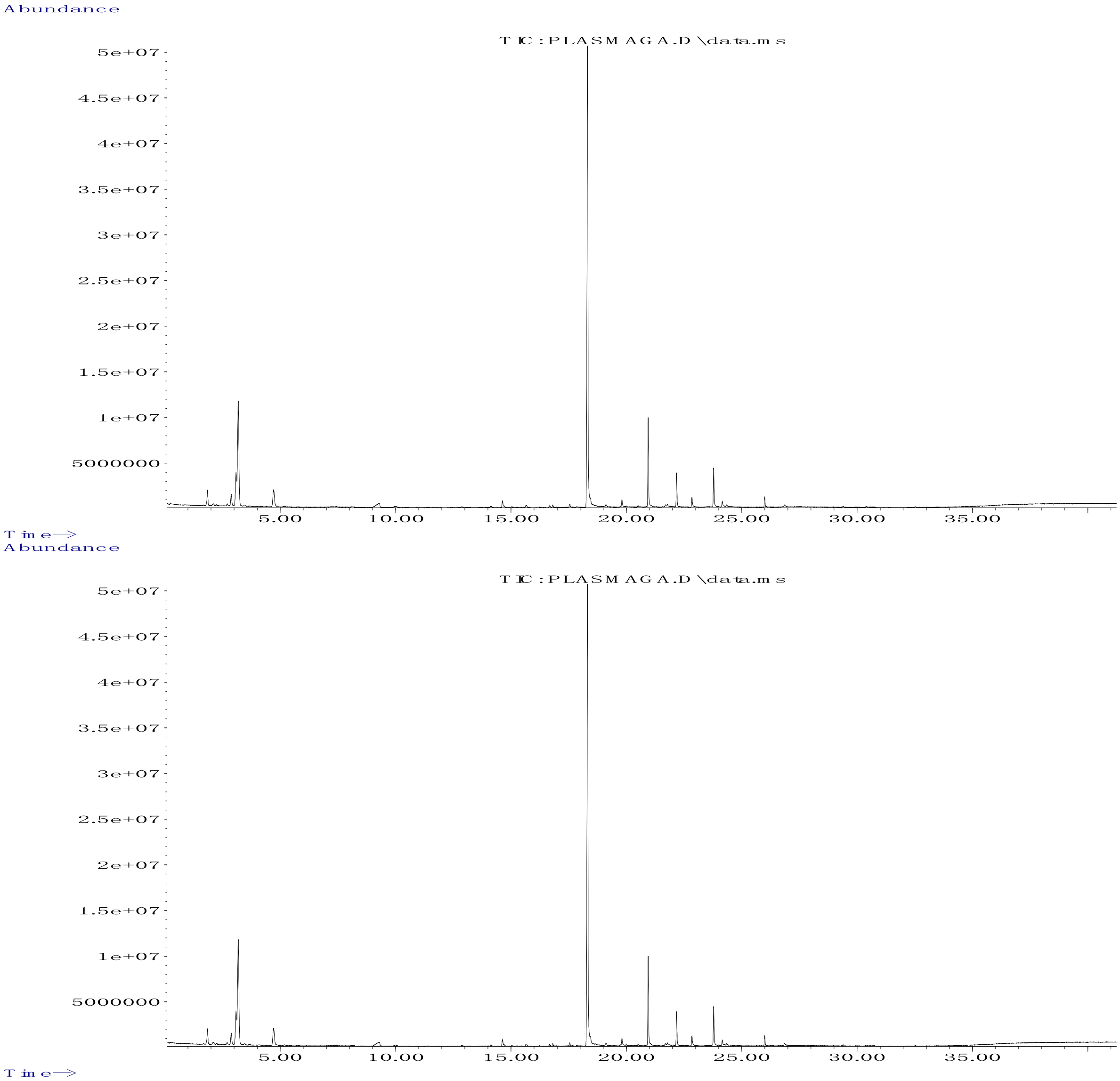

Figure S1. Chromatograms showing the volatile composition of hydrolysates of **A** minced bovine meat (BEEF), **B** bovine heart (HEART), **C** porcine hemoglobin (HEMO), and **D** porcine plasma (PLASMA) in dublicates (A and B) with or without glucosamine (G).
